# Mixed methods, mixed feelings: a review of hurdles faced and vaulting poles to apply when wanting to do and publish mixed methods research

**DOI:** 10.1080/1369183X.2025.2487748

**Published:** 2025-05-17

**Authors:** Niels Spierings, Nella Geurts

**Affiliations:** Department of Sociology, RSCR, Radboud University, Nijmegen, the Netherlands

**Keywords:** Mixedmethods research, ethnic and migration studies, publishing

## Abstract

Sometimes ‘mixed methods designs’ are considered a winner for obtaining research grants, but a close-to-certain reject when publishing. Evidently, reality is more complex. At the same time, these considerations are grounded in actual experiences, observations and the structure of our epistemic community. In this contribution, we will reflect on this structure, particularly in ethnic and migration studies, as such reflection is particularly interesting for a case that is strongly interdisciplinary, which might pave the way as well as lead to double jeopardy (running into reviewers disliking the method and the disciplinary perspective). First, we will sketch a (quantitative) background of the definition and prevalence of mixed methods research in ethnic and migration journals. Next, and based on migration scholars’ experiences with mixed methods research and often heard ideas, we will chart and discuss the structural barriers that hamper mixed methods research (from journal word counts to unqualified reviewers). Third, based on these barriers or hurdles as we will label them, both structural and practical solutions are discussed, leading to a wish list for facilitating high-quality mixed methods research.

I searched the journals on the term ‘mixed methods’,

the website gave a total error and my laptop almost took fire.

## 1. Doing mixed methods research successfully?

During the research for this contribution, one of us sent the other this text message and we had a good laugh, considering this is symptomatic for the state of the field. But, laptop overheating can imply either that it was totally confused what we were looking for, if the term ‘mixed methods’ was rather alien to the search engine, or that there were so many returns on this search input that the machine simply could not handle it. Which one of the two is most representative of the state of the field, is one of the questions answered in this paper.

To be upfront, we start from our own observation as sociologists that mixed methods research is often supported as an idea while it remains relatively scarce in practice. Here, an often heard advice is not to conduct it because it is hard to publish (although it is sometimes considered a fashionable way to obtain grants). At the same time, we started the process of collecting input for this contribution with a wish to be surprised. And with that in mind, we set out to provide an overview and an understanding of mixed methods research in ethnic and migration studies through the lens of (fellow) researchers in the field. This understanding extends to critical discussion of the hurdles mixed methods research (plans) run into, as well as the potential vaulting poles that can help jump those hurdles, and what this implies for the different actors involved (i.e. authors, project leaders, supervisors, editors and reviewers). As such, this study has three aims of which the results inform each other: (1) obtain how mixed methods research is defined ‘on the ground’, (2) establish to what extent mixed methods research has (mixed) success in practice and (3) explore which hurdles to overcome and what vaulting poles are present among researchers (not) doing mixed methods research in ethnic and migration studies to do so. As perhaps clear by now, we apply metaphors relating to i.e. hurdles and vaulting poles to make the various puzzle pieces that are part of the mixed methods research track more tangible. As such, we hope that these metaphors produce useful mental images of experiences as well as tips helpful in reaching the finish victoriously when doing mixed methods research. And at the same, now having written this contribution, we realise this metaphor echoes that academic reality is (partly) a competition, and that turns out to be a hurdle in itself.

To be entirely clear, this article is not a methodological treatise to explain why the application of mixed methods is important. By now, many have convincingly argued that (good) mixed methods research brings something new to the table, something that transcends the sum of its parts, answering unique questions and providing novel insights (Bergman 2018; Horvath and Latcheva 2019), including authors in the area of ethnic and migrations studies (and this journal, specifically) (Kondor et al. 2022; Yeung and Mu 2020). Similarly, quite a number of methods, textbooks and articles provide principles and guidelines for doing mixed methods research (Gobo 2015; Schoonenboom and Johnson 2017; Small 2011; Tashakkori and Teddlie 2008) and other contributions in this Special Issue add important and fascinating new insights to those, often highlighting the murky and tricky practices involved (see for example Merlín-Escorza 2025).

We assume that our readers are open to mixed methods research and fully trust that you will be able to conduct it or learn to do so. However, from many scholars we have heard that they run into issues conducting and/or publishing it. Others, particularly young scholars, have confided with us that they were considering conducting mixed methods research, but have been advised not to. And some do not feel like conducting mixed methods research themselves, but are wondering how they can further and support the publication of mixed method research because it does inform their work nicely or are supervising PhD candidates who want to mix methods. For all these scholars, we hope that reading this article provides some recognition, joy, practical support, advice and suggestions for future success.^1^

This contribution started from our own personal experiences with applying, doing and trying to publish mixed methods research. In line with the set-up of this special issue, we will build on those experiences. However, we would not be mixed methods researchers if we did not pull in some other ways of gathering information too. We surveyed several ethnic and migration studies journals that triggered all kinds of ideas and debates on what mixed methods research is and what shapes it can take. A part of the answers to such questions was also derived from an online conference we organised in October 2021, a short qualitative survey we conducted among two ethnic and migration scholars networks at the end of 2022, and an informal focus group with ethnic and migrations scholars organised in our research institute in January 2023.

As is always the case with mixed methods research, the challenge in this article will be to bring the parts together; here these are all our own experiences, the survey data, the review tally and group-interaction data together. We have set out to do so in an integrated and meaningful way: below, we provide three substantive sections (and a closure section), each of them drawing from multiple of our data sources.^2^ First, we provide insight into what actual ethnic and migration scholars consider to be mixed methods research and to what extent it is conducted and can be found in core journals in ethnic and migration studies. This grounds our further discussion in actual practice. Second, we delve into the (perceived) hurdles to doing mixed methods research, including a critical discussion of the provided arguments. Only by collecting a broad range of hurdles can we start thinking about what is needed to jump them, which is exactly what we will do in the third section. Considering the hurdles identified and what academics indicate to need, we will start formulating possible ‘solutions’ for different actors on more shorter and longer terms, of course, not without ignoring the context of academia today.

As already mentioned, this contribution sets out to provide solace and practical suggestions spanning the entire trajectory of doing mixed methods research and making it a success. Moreover, we hope this article to contribute to a larger discussion and some rethinking of our practices in the many academic roles we take. As discussed in the Introduction of this Special Issue (Geurts, Davids and Spierings 2025), we will do so for the case of ethnic and migration studies (EMS). In EMS, paradigms and disciplines – and associated expertise, tools and methods – meet to further our understanding of the migration and inclusion processes. As such, we expect EMS to be a mixed methods frontrunner in the social sciences, being a valuable case to learn about the best practices and develop our thinking on mixing methods. Although we have little reason to assume that the hurdles we identify below are unique to EMS in their nature, concrete actions for steps forward always need to be formulated acknowledging the specific context (i.e. supervisor, department, institute, country and discipline).

## 2. Sources of data mixed for this study

As said, we have analysed five data sources to provide insights in our three research aims. Throughout our analyses, we mix these data sources to offer a more comprehensive picture of the issue at stake.

First of all, the foundations of this contribution lie in a **virtual conference** we organised, together with Tine Davids, on Mixing Methods in Migration Research on October 21st and 22nd in 2021. The conference included two keynote speakers, Jørgen Carling and Rossalina Latcheva, as well as several workshops on the practices around mixed methods research. The insights shared during the conference (from speakers, participants and discussants) and the notes taken during the conference, including a 21 page report on it,^3^ are built upon in the analyses.

Second, we collected **data from ethnic and migration journals** to explore how many articles used a mixed methods design and how these articles perform when looking at some metrics available. The eight journals provided in Table 1 were selected on being mainstay and well-renowned journals in ethnic and migration studies, spanning a broad scope of disciplines, for instance covering demography, sociology and ethnic studies as Clarivate discipline categories. From each journal, we searched all original articles (i.e excluding reviews, book reviews etc.) on being self-identified as mixed methods, based on variations of the search string [mixed method]. For each, we checked whether this referred to the method applied in the study, not a suggestion for further research or a study being referred to. Except for JEMS, The time scope was limited to everything published in between 1/1/2020 and 31/12/2022. In addition, we checked the metric scores of these articles compared to published articles with a similar age in the respective journal.

**Table 1.** Mixed methods articles in 8 ethnic and migrations studies journals.

Journal (2020-2022, unless stated otherwise)	Self-identified mixed method articles	Total number of articles	Percentage mixed method articles	Classification	2021 Jif percentile	2022 Volume number
Journal of Refugee Studies	15	253	5.9%	Demography	58	35
				Ethnic Studies	71	
Journal of Immigrant & Refugee Studies	9	170	5.3%	Demography	79	22
				Ethnic Studies	76	
				Sociology	71	
Journal of International Migration and Integration	15	321	4.7%	Demography	51	23
Journal of Ethnic and Migration Studies	23	557	4.1%	Demography	65	48
				Ethnic Studies	92	
*2015-2017*	*6*	*512*	*1*.*2%*			
*2010-2012*	*2*	*248*	*0*.*8%*			
*2005-2007*	*1*	*190*	*0*.*5%*			
Comparative Migration Studies	6	158	3.8%	Demography	81	10
International Migration Review	3	186	1.6%	Demography	75	56
Ethnicities	3	187	1.6%	Ethnic Studies	24	22
Ethnic and Racial Studies	6	430	1.4%	Ethnic Studies	55	45
				Sociology	57	
Total 2020-2022	80	2,262	3.5%			

We, moreover, collected data via **a survey with open-ended questions** among two ethnic and migration scholar networks in the Netherlands (RUNOMI: Radboud University Network on Migrants and Inclusion; DAMR: Dutch Association of Migration Research), in the period between October and December 2022.^4^ Our 24 respondents answered questions on their definition of mixed methods, their experiences and perceived hurdles (see Appendix A for the questionnaire). In the survey invitation, which was shared through the general e-mail address from each network, we explicitly invited scholars who did and *did not* have experience with mixed methods research (and even those who hated this type of research).

After the first analysis of these survey data, we discussed and raised follow-up questions in an **informal focus group** with five fellow ethnic and migration scholars in our research institute on January 26th, 2023. The participants included more junior and senior scholars, generally with limited experience in doing mixed methods research and being trained mostly in quantitative methods. During the focus group, we asked them what struck them based on our analyses of the survey data and talked more in-depth about their considerations and aspirations in doing or not doing mixed methods research.

And last, we throw **our own experiences** in the mix. To situate these experiences, let us shortly introduce ourselves and our experiences with mixed methods research in ethnic and migration studies. I, Niels Spierings, Professor of Sociology, have been trained in both qualitative and quantitative methods and have worked in departments dominated by either one or neither. As a result, I think I have a rather broad comprehension of the different languages involved, and this probably turned me into a bit of a methods geek. As such, I never really understood the antagonisms between approaches and I like drawing from different literatures. At the same time, in my experience, manuscripts based on multiple or in-between methods are relatively hard to publish. Having said that, it is also these articles that are more likely to be praised or win prizes once published. Mixed success, indeed.

I, Nella Geurts, am an Assistant Professor in Sociology with a specific focus on doing mixed methods research when studying migrants’ inclusion. As a Bachelor student in Sociology, with a focus on developing quantitative research skills, I already caught an early interest in qualitative methods due to both parental and roommates’ socialisation, who had a more anthropological background. As someone who is always looking for the best of both worlds, the realisation that a mix of methods could be fruitful was sparked after a discussion with roommates at the kitchen table on how to best study migrants’ ‘integration’. After my Bachelor’s, I fed this interest by attending various courses and a summer school on mixed methods research and, eventually, put it in practice in my PhD thesis on the integration paradox. I count myself lucky that these mixed methods endeavours were seemingly appreciated by journals and reviewers who accepted articles using such a design, and these skills were valued in academic positions that I applied for. Nevertheless, as someone who loves discussing these designs with others, I notice and am aware of the (practical and structural) struggles that are faced by researchers (including me) when considering such a research design.

## 3. Mixed methods in current ethnic and migration studies

What is considered to be mixed methods research and do we, EMS scholars, conduct and publish it? Answering this (seemingly) straightforward question provides a glance at the discourse we have been practicing in. Moreover, doing so also lays a fundament for future researchers: the answer will lay bare a (concentrated) wealth of mixed methods research (MMR) in EMS, even though, in relative terms, MMR is as scarce as Turkish migrants in Germany.^5^

### 3.1. What is considered to be mixed methods research?

The dominant, if not omnipresent, lived definition of mixed methods research among ethnic and migration scholars is one of mixing quantitative and qualitative methods.^6^ In our survey data among Dutch EMS scholars, 18 of the 24 substantive answers to the question how they would define MMR explicitly referred to combining quantitative and qualitative methods, mostly as part of the definition and, in some cases, as single illustration of an akin formulation like ‘methods from multiple epistemological backgrounds’. The other six definitions do not refer to specific methods, but do include conditions such as ‘substantially [different]’, ‘particularly (…) unusual mix’ and ‘from various disciplines’. Moreover, when these respondents provide MMR examples from their own work, if any, they are often a combination of qualitative and quantitative work too.

That ‘mixed methods research’ is interpreted as qualitative-quantitative work might, of course, be an artefact of the Dutch academic context and our small sample size. At the same time, Dutch academia is very much oriented to (quasi) international (i.e. European and Anglo-Saxon) academia and our findings very much resonate with what we found reviewing the 2020–2022 publications in eight leading EMS journals: the vast majority of self-identified mixed methods studies presented a combination of quantitative and qualitative research.

Evidently, there are deviations to this dominant lived definition, which shed some additional light on the discourses surrounding MMR. First, the self-defined MMR articles that are not of a quantitative-qualitative cut are almost exclusive studies combining different qualitative approaches (often in terms *triangulation* to get closer to the truth – as positivist researchers may put it – or *crystallisation* to uncover and co-construct multiple truths – as interpretivist researchers may put it (Ellingson 2009)). Also, in our discussions on the special issue, the issue of intra-qua[nt/l]itative MMR was brought up, referring to qualitative MMR (see Davids, van Houte, and Alpes 2025; Merlín-Escorza 2025) and, to a certain extent, quantitative MMR (see Hofstra 2025). Among survey participants, it seems that researchers conducting work that can be considered quantitative MMR feel less of a need to use the term ‘mixed method’. Does this imply that quantitative researchers are looking less for methodological labels to justify their mixing approach?

Second, in line with some of the seminal works, several of our surveyed colleagues stress additional conditions for multiple methods to be considered MMR: ‘build on each other’, ‘to provide answer to *one* research question’ (emphasis added), ‘to speak to each other’, ‘combine findings’ and in alluding to the whole being more than the sum of its parts. These answers reflect the distinction between multi-methods and mixed methods research (see Plano Clark and Ivankova 2016) — the former referring to ‘1 + 1 = 2’ and the latter to ‘1 + 1 > 2’. Based on our focus group, we also see that many who simply wrote something about combining stuff actually also implied that at least the outcomes of the separate and methodologically distinct analyses should come together at some point, a rather pivotal point when thinking through the future of mixed methods research.

So, EMS scholars tend to refer to MMR as work integrating quantitative and qualitative research, and this – our third and final observation on defining MMR – seems mutually constitutive with a certain connotation. Mixing quantitative and qualitative research is considered to bridge epistemological boundaries, which has negative connotations for many. In the survey and focus group, references were made to Babylon, antagonism, conflicting perspectives, fights, a lack of respect and disloyalty. However, these elements are not just considered hurdles to overcome for MMR. No, they are actually also mentioned more generally when defining and describing MMR: the bringing together of conflicting views has become innate to what mixed methods research is, at least for some, but certainly not rare exceptions. In this realm, respondents also refer to paradigms that view either qualitative or quantitative methods as superior, which ties with the so-called knowledge hierarchies (St. Pierre 2013): a hierarchical difference in the valuation of specific types of knowledge. Such a hierarchy affects viewpoints that supervisors and journals may have with respect to mixing methods. In various ways, hierarchical thinking trickled down in experiences shared. Explicitly, some saw more value for a certain paradigm than another, and implicitly, by acknowledging that *adding* a few interviews would shed some more light on the existing quantitative findings, attributing more value to the latter, or the other way around that adding a few general statistics is useful as a springboard to show how this veils important nuances.

In brief, and slightly simplified, mixed methods research has two faces. Theoretically, it is a neutral combination of different methods that are integrated in applications or output, so that its analyses transcend the sum of its parts. Empirically, mixed methods research (in ethnic and migration studies) is predominantly the attempted combination of qualitative and quantitative methods that are integrated in applications or output so that its analyses transcend the sum of its parts, when successful, but that is not a given.

### 3.2. How much mixed methods research exist and where do we find it?

The discussion above already alluded to the findings of our scoping review among EMS journals, but how many articles in these journals are self-identified mixed methods research?

As Table 1 shows, over three years’ time, the 2,262 articles published in eight different reputed journals included only 80 self-identified mixed methods studies, that is 3.5%. Whether this is much or little depends on one’s perspective and expectations; nevertheless, a bit more can be said on this figure. First of all, it really does seem to depend on the journal, with the current variation running from 1 in 72 to 1 in 17 published studies being self-identified MMR. Second, our review already leads to a list of 80 articles in EMS that could serve as examples (more below; the online material contains a bibliography). Third, our over-time comparison conducted for JEMS suggests a steady and even accelerating increase in self-identified MMR.^7^

Moreover, while we cannot make claims about the (relative) success rate of the submitted MMR studies, our survey, for which we very explicitly invited EMS colleagues both when they loved or hated mixing methods, provides some of the first insights on whether people conduct *and* publish MMR.

Our colleagues sketch a picture that nuances the above somewhat. Of them, 16 of 25 (65%) said to have conducted MMR research: 14 (56%) qualitative-quantitative and 3 (8%) (also) qualitative mixed. And 32% (8 of 25) has published MMR in journals, edited volumes or monographs; or 28% when only qualitative-quantitative designs are considered as MMR. As our question referred to ever (in their careers), evidently not all their research has to be MMR. Still, these percentages align more with the idea that EMS might be particularly fitting to mixed methods, given the already interdisciplinary, boundary ascending character of EMS.

**Figure 1.**
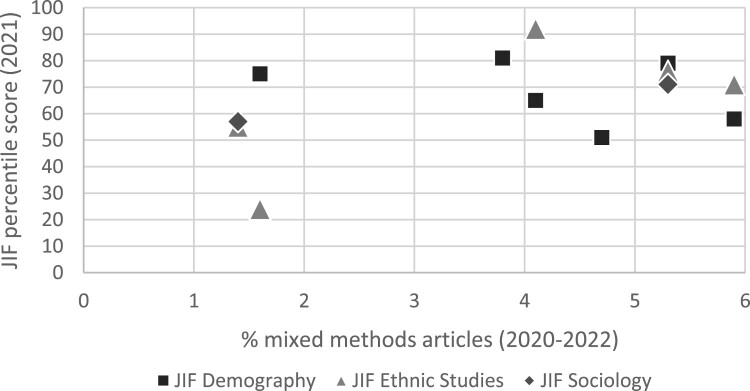
Relationship between JIF score and publishing mixed methods research.

Taking one more dive into the prevalence data in journals, we apply a comparative perspective to grasp the dynamics a bit more. Table 1 shows that the eight studied journals are classified in three disciplines (by Clarivax). However, there does not seem to be a clear pattern that journals in a certain discipline published more or less MMR studies. Next, we compare the (citation-based) JIF percentiles of the journals with the prevalence of any type of MMR in them (Figure 1). At first sight, there is no clear pattern to this either, except that publishing at least 3.8% mixed methods articles seems sufficient for a JIF score above 50, which does not hold for the less MMR-minded journals; however, the latter is fully based on one journal (Ethnicities), so we should be cautious here.

If anything, our data on the eight journals suggest two potential patterns that deserve further study and reflection. First, a weak relation seems to be present between the age of the journal and publishing MMR, whereby the newer ones are seemingly more likely to publish MMR. More particular, three of the four journals below the age of 24 pass the 3.7% bar while three of the four journals aged 35 and up publish fewer than 1.7% MMR articles. Second, Table 1 also shows that the refugee studies journals, which arguably lean somewhat more towards law as a discipline, publish more MMR. Accordingly, also in our survey, the five respondents whose primary position was in the discipline of law all indicated to conduct MMR (against 65% overall) and three of the five conducted quantitative-qualitative MMR (the other two mixed qualitative methods). Altogether, this suggests – but let us not overstress the meaning of this – that MMR might be relatively more common in the discipline of law, which can be (partly) attributed to a broader definition being used in law compared to the more dominant quantitative-qualitative focus in other disciplines.

### 3.3. How else do mixed methods manifest themselves (invisibly)?

In the above, we delved into the perception of what a mixed method is and how prevalent it is in (published) research, both within ethnic and migration studies. Our review, survey and additional sources also lay bare or hint at other manifestations of MMR, which deserve particular note for fairly assessing the hurdles in (Section 4) and future of (Section 5) MMR in EMS.

Talking about the visibility and integration of MMR in our field, research is more than original research journal articles. Importantly, our journal search reminded us of the importance of (scoping, systematic, literature, narrative or integrative) **review articles**. We came across quite a number of these, explicitly including mixed methods research as part of their scope on topics as diverse and specific, as migrant home care workers (JIMI), labour market discrimination against ethnic minorities (JEMS), refugee trauma (JIRS) or internally displaced people in Africa (JRS). This is particularly important in terms of acknowledgement and visibility as review articles serve as a gateway to other research on specific topics and as agenda-setting for future research.

Next, while in our own work and collaborations we often distinguish between mixed methods publications and mixed methods projects – the latter is particularly helpful in offering avenues for advancing MMR – we were surprised how often this was also addressed by other scholars (see Section 5), particularly in journal articles. We found around 20 journal articles from at least six (out of eight) journals that reported on mono-method research, while explicitly noting that the research in the article was **part of a larger mixed methods project**. We deemed this particularly interesting, partly because it never came to our mind to do so in our own mono-method articles from a mixed methods project, not the least because we often end up having to trim a couple hundreds if not thousands of words from the manuscript, and non-crucial information like this is then sacrificed. In other words, there might be quite a number of mono-methods articles embedded in mixed methods research, which we might never know of, suggesting our estimates here are on the lower bound of MMR’s prevalence.

Now, we are at the theme of MMR being conducted but not being published as such, and our survey also provides some telling insights. Of our colleagues, 65% (16 of 25)^8^ indicated to having ever conducted MMR; however, only eight of them (50%) report to have published this research in scientific outlets: journals or books.^9^ This suggests that MMR exists and is conducted, while **never making it to scientific print**. Considering our own experiences with the degree of research that leads to publications, this percentage seems considerable, although this conducting MMR might, thus, also refer to projects instead of journal articles or book chapters. Yet, if so, this does raise the issue when a project can be considered as *mixed* methods. Where and when are the different methodological strands in the project integrated (see Section 3.2)? Also, articles that mention being part of a mixed methods project say surprisingly little about that part of the mixing, an issue which we discuss more in Sections 4 and 5.

Another way in which MMR was mentioned in articles in a seemingly positive way is suggesting that others should **do some mixed methods work in the future**. However, this was actually less common than we expected. We found 12 examples of this covering 6 of the 8 journals, often included at the end of an article in which the authors themselves did not report on MMR. Although some might consider this lip service to reviewers – yes, we have heard those arguments too – these suggestions should not be discarded too quickly. Particularly, if formulated well and concretely (which is certainly not always the case), such remarks might inspire others and equip them with references and quotes to convince funders, supervisors and reviewers.

Finally, although we have fairly little information on this, we want to raise **the issue of visibility and impact of research**, and MMR specifically, more broadly. One survey respondent explicitly referred to the use of MMR in reports for government, which was also stressed in the conducted focus group. From our own experience, we also know there is a broader interest, as voiced via invitations for key notes, presentations and informal advice. If anything, this signals that there is a need for (hands on) information – which came to the fore in several workshops during the mixed methods conference too – and this was especially voiced among junior scholars.

**Figure 2.**
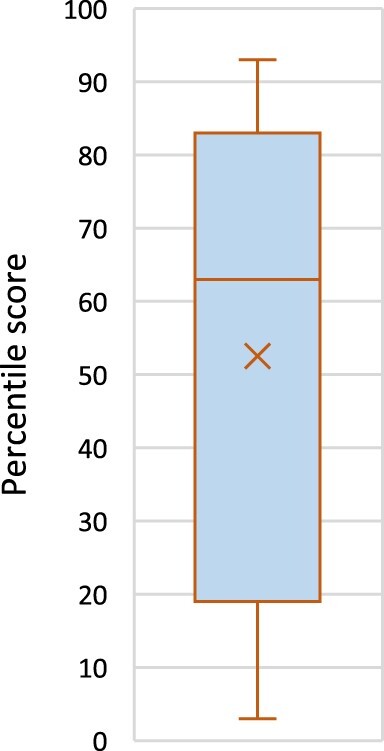
Impact metric for JEMS 2020-2022 articles.

In extension of the above, we also took a quick snapshot on the impact metrics of the articles in JEMS compared to all other articles in JEMS of the same age (as provided online), which is based on articles being referred to online in news coverage, policy documents, blogs, Wikipedia and social media.^10^ The results are summarised in Figure 2, whereby the X-marker indicates the average score and the middle horizontal line indicates the percentile score of the middle ranked article of the 23 included. If the MMR articles are equivalent to other articles, the mean should be at 50 and the boxes and whiskers of the box-and-whisker plot should be symmetrical around the 50 percentile line. Particularly, the latter is not the case. The mean percentile is 52, but for every MMR article below the 50th percentile, we found over 1.5 MMR articles above it. Again, we should be very careful here, but these are indications that mixed methods articles might attract some more attention (which does not mean that this is *because* they use MMR).

## 4. Hurdles

The discussion on published/ing mixed methods research in EMS (Section 3) and the seemingly discrepancies between the degree of conducted and published MMR provide first indications that our own experiences about the existence of (perceived) hurdles are shared more broadly. Also, the material suggested that doing and publishing MMR might be tougher in older journals, in disciplines not being law, when one does not have a permanent position and because of the (perceived) conflicting nature innate to combining methods with different epistemological roots. Our survey^11^ and other qualitative materials put more flesh to the bone of this idea of hurdles. Based on those data, this section will provide a more systematic overview of (potential) hurdles, a necessary step for thinking about ways to overcome them and thus facilitate the growth of knowledge with insights that can only come from MMR.

Table 2 summarises the arguments collected, indicating which were the most dominant and grouping them in terms of the locus of the hurdle. Evidently, this is an analytical and heuristic classification as the loci are interrelated. The overview is not a list of truths in the positivist sense; it includes personal truths, discourses, perceptions, experiences and realities. Below, we will not reiterate the list, but we will draw out patterns and observations relevant to the larger question of this contribution.

**Table 2.** Hurdles and vaulting poles to conducting mixed methods research.

Locus	(Perceived) hurdles	Vaulting poles
MMR (‘in-nate’)	The jargon or language of methods differ, making it hard to let parts really mix, to make them speak to each other. It leads to more shallow inquiries. Surveys cannot be personalised to fit such qualitative data, which makes sequencing the parts problematic. One of the methods has to be leading, so the other(s) will be reduced to second-order forms of research	Respect and appreciation for all methodological corners and knowledge that specific research questions benefit from different methods used, regardless of interest in conducting it as a researcher Facilitating conversations between researchers on similar themes using different methodologies, with goal/assignment to learn from each other
Researcher	Fear of research not getting published **Researchers are only trained in one (set of) methods. Not having the skills, knowledge or experience to apply the other (set of) methods well and comfortably. Or at least not feeling equipped to do so.** A lack of knowledge of what possibilities or methodological options exist. A lack of knowledge of doing MMR: how to mix, how to sequence, how to weigh results of different methods, how to combine different languages. Fear of reputational damage for engaging with an inferior form of doing research or for presenting just a trick or showing off. Disliking, not enjoying of being prejudiced against ‘the other’ method.	(Accessible) examples of mixed methods research to offer inspiration and tools in, for example, a database clustered by a theme or design (Accessible) mixed methods training, especially for PhD scholars
Other researchers	Not being taken seriously by collaborators with different expertise who consider their method best or even the only proper method and other epistemological clashes, all of which might lead to projects falling apart, not succeeding. Hard to find a co-researcher with whom this could work or specifically a person who is versed in combining methods, languages and perspectives. The required teamwork implies a certain loss of independence	Collaboration database with those expressing their expertise and/or interest in conducting mixed methods research on specific themes (to enable cross-methods connections).
Structural context	Investing in MMR research and publishing is not valued as much as monomethod research and you might be considered no expert in either method, making it a bad career choice. **Epistemological barriers and antagonism discourage, take away or nullify the joy of doing research.** It simply does not dawn as an option given the existing divides. Given divides and language differences, it is hard to reach multiple audiences with MMR. Where I work, there is no one to bridge between methods. **In (current) academia, there is no time for more time-intensive research like MMR, to learn new methods, to develop new tools and design. I just do not have the time.** Long-term projects require a proper budget, also budget covering multiple modes of data collection. Funders need to be convinced of the added value of spending that kind of money. **Useful secondary data for MMR is limited: sharing or using secondary qualitative data is fairly uncommon and by some considered difficult if not impossible; existing quantitative data is often considered ill-fitting with the goals of MMR; quantitative data often do not include a large enough subsample of hard to reach populations or minorities, which are often the focus of qualitative EMS scholars; and in many parts in the world access to secondary quantitative data is problematic.** Dreaming big is not taught or valued: we think in small paper-size questions, not going for the larger issues, the overarching questions we want to answer. We start small and sell it as bigger instead of starting big and breaking it down to smaller bits and pieces.	An open academic atmosphere in which collaboration and team science is applauded as well as learning about new methodological approach. Sharing (both qualitative and quantitative) data that have been already collected openly within the academic community. Sharing data more would underscore that MMR does not necessarily require one researcher to do it all.
Journals, reviewers and editors	**It is difficult to convince multiple audiences and the chances are relatively high that reviewers will misunderstand or hate at least half your paper. Reviewers are allowed to review parts of your work they have little knowledge of and they do.** Journal editors do not take responsibility in judging the reviews, are not knowledgeable themselves or even prejudiced. mixed methods studies are not valued by the scientific community; it is only lip service. **Word count restrictions are killing and becoming ever more lower, while MMR implies you need to explain multiple methods and data sources as well as relate to multiple literatures, which is impossible when only 7000 or 8000 words are allowed.** **It can be hard to find a journal that is open to MMR, because many journals are geared towards a certain method or (monomethod) discipline. Often it is unclear whether a journal is open to MMR or whether it understands and accommodates particularities of MMR. I do not know what journals to submit to.**	As a journal: making explicit whether mixed methods research is welcome and have editors with expertise in this matter. indicates to what extent using more words is allowed and whether using a mixed methods research design is a valid reason to do so, or make more extensive use of online appendices. support project-based notes that prior mono-method articles are embedded in, making MMR more visible and stimulating proper integration of methods. As a journal editor: invite reviewers with mixed methods experience or instruct reviewers who do not have that to focus on the methods they have expertise in.
(PhD) supervisors	PhD students are not or should not be allowed to conduct MMR, because the risk is too high, the sequential design takes too much time, and the career perspective is dire. Supervisors are not (considered) capable of supporting PhD students with certain methods or not capable of working together with experts from the other side (even if they are supportive of the idea). PhD students do not feel comfortable suggesting it because of anything supervisors have voiced between hesitancy to dismissiveness on MMR.	Stimulate an open academic atmosphere in which collaboration and team science are applauded as well as learning about new methodological approaches Enable collaborations with researchers with other methodological expertise and funding for PhD scholars to get training in mixed methods research (of course, depending on candidate’s preferences)

Notes: Text in **bold**: the most prominent and widely voiced hurdles

Sources: Survey among RUNOMI and DAMR members, Conferences (see section 1), MINI seminar Nijmegen datum sociology, personal communications and observations from authors.

### 4.1. Hurdling

Some observations relate to the entirety of the collection of hurdles. First, filling a big table turned out rather easy, but noteworthily, we got many more answers on the question about perceived hurdles (i.e. not per se referring to one’s own choices), than on the question on one’s own reasons for not doing MMR. It seems some academics avoid MMR because of expectations. In terms of our analogy, they never step onto the track to start their hurdle race.

Second, there is a reason for why we use the analogy of hurdles instead of talking about ‘walls’^12^ or barriers. As the overview in Table 2 shows, there is a whole sequence of issues that can prevent one from conducting and, in the end, publishing mixed methods research. Once you jumped the first hurdle (e.g. coming up with the idea) there are many more to come (e.g. convincing your supervisor, securing resources, condensing it to 8,000 words, satisfy reviewers, etcetera).

Third and relatedly, taking all these hurdles together, not conducting MMR seems to be a function of at least three conditions, each of which is independently sufficient to prevent MMR from reaching the end of the racing track: *not being able to*, *not wanting to* and *not considering it opportune*.

### 4.2. The hurdles

Turning to the specific groups of hurdles, we draw out some patterns and provide reflections. First, the elements we classified ‘*innate’* could also be considered as a part of the structural context. However, the elements mentioned as part of the innate were, in our interpretation, presented not so much as contextual but rather as fundamental. The ideas were not ascribed to others, but are convictions of individual researchers, which prevent any desire to conduct (any type of) MMR, even if good opportunities arise.

At the *researcher* level, lacking expertise was very often voiced as a hurdle to overcome, which deserves some unpacking. This lack of expertise has more than an objective side: the subjective side includes confidence and self-efficacy. It also refers to three different phases: expertise to select fitting methods, expertise to apply ‘the other method’ and expertise to mix methods. Moreover, and interestingly, implied in many comments was that research is an individual endeavour, implying that one person needs to have all expertise. Finally, experience and expertise were used interchangeable it seemed, while to us, this is an important distinction particularly in light of training and looking beyond solo research.

Considering collaboration with *other researchers*, the antagonism and existing convictions that specific paradigms (and associated) methods are superior to the other came up again. At the same time, also mentioned is the need for people who are versed in multiple (and mixed) methods, people who can tie elements together or functions as interlocutors. They seem rare, regardless of whether institutes are mono- or multimethod.

Our fourth category– the institutional *context and structures* at large– came up a lot; an important observation in itself: even if researchers want to conduct mixed methods research, they are discouraged by practicalities like a lack of fitting data, no time, no money, and no recognition and reward. A burning question here is to what extent these expectations that discourage people would actually manifest themselves once taking the step to conduct MMR. The structural context might not be conducive to MMR, but a part of this story is also that this might be a set of partly misleading perceptions, and such perceptions can be overcome differently than the structures itself.

Zooming further in, despite that we found a considerable number of mixed methods studies, *reviewers, journals and editors* are often mentioned as part of a discouraging context too. This involved practical issues – word counts; lacking knowledge on which journals are open to MMR – and issues more intertwined with the elements discussed above, such as having the skill to talk to multiple audiences and antagonistic reviewers one might run into.

And finally, the role of *supervisors* should not be ignored, but this did not come up as much as we expected. A part of the story here can be the settings and the limited number of PhD respondents in our data collection. Still, supervisors themselves indicate that they seem hesitant to allow PhDs to involve in MMR, often grounded in trying to do the best for the careers of the PhDs.

All these hurdles together seem to produce a rather toxic cocktail of ‘want not’, ‘cannot’ and ‘need not’. Academics consider doing (especially QQ) MMR a pain they do not want to be involved in. Might they actually consider it fun or useful, it is considered partly impossible because of their own incapacities or the lack of experts, data, money or permission. And when that is solved, the ‘output cannot’ looms: it will be rather tough to get published. Finally, initially accepting this hardship, the zero-sum consideration is made: ‘Do I want to invest in this, which implies major investments and low(er) expected pay offs even if published? No, as I need not, in order to further science or my career’.

### 4.3. On the hurdle

Before turning to solutions (Section 5), we want to take one step back and offer some additional reflections on the above, also bringing the information so far together, particularly so because we are on the fence of some issues: the reported hurdles and the registered successes seem contradictory.

‘Want not’, ‘cannot’ and ‘need not’ are for certain real in terms of their impact, as they are experienced. At the same time, our personal and others’ experiences do not always align with these seemingly persistent perceptions. We did manage to publish mixed methods research, and, in the field of ethnic and migration studies, the process has been particularly smooth compared to other fields and disciplines. Concurrently, however, we should realise that publications might have been rejected by many other journals before. Unfortunately, we have no information on how many MMR manuscripts have been submitted and what their (comparative) success rate is. Nor do we know whether projects entailing MMR produce relatively more books, of which the format is less restricting, at least in our experience.

Also, a recurring theme was antagonism. While methodological antagonism still exists, it partly seems to be an echo from the past. As sociologists, we hope and expect intergenerational replacement to lead to attitudinal change in that respect. Moreover, based on all our data collections, we think there is a pattern in the development over time. First, there was no communication between scholars using fundamentally different methods (i.e. quantitative versus qualitative). Then, there was fierce battle and antagonism. Now we seem to shift towards peaceful coexistence or lip service, stating that mixing methods is valuable and important without necessarily being involved in it. We consider the latter necessary and a step up from antagonism, but not sufficient, for further development of mixed methods research. If we look at this trend, we hope that respect can be next, followed by integration. This implies that not only there is some work ahead, but also quite some distance has been covered already. Evidently, change does not happen by itself, but rather is brought about by structural initiatives and vaulting poles that are introduced and enable such change over generations, such as in how methods are taught and what editors value in journal articles.

Lastly, surprisingly little came up about grant applications, neither in terms of ‘mixed methods’ being a buzzword, nor it being impossible to get MMR funded. Our own experiences in this respect are mixed, but we have little indication that it differs from grant applying in general: your approach needs to fit the questions and speak to the audience. If so, this implies MMR hardly forms a risk or strength, as long as you know your business.^13^ But as always, we depend on reviewers, editors and committee members. To illustrate, for one (in the end successful) application, one of us received the following comments, both praising the mixed methods approach and acknowledging a typical hurdle:

The methodology is sound, tightly related to the research question and the added value lies particularly in a) clear operationalization of theory in items b) the mixed methods and c) the tightly connected phases – quantitative contextualization.

I think this motivation to go beyond disciplinary and methodological boundaries and specializations is an important asset.

The plan for the qualitative data collection (interviews) seems underdeveloped (…) It is possible that the word count did not allow to be more specific on these points,

## 5. Vaulting across the mixed methods track

Having identified the hurdles that scholars perceive and run into, let us consider what can help overcoming them: the vaulting poles (mixing two athletic techniques in our analogy, being quite sure vaulting poles will get a professional over a hurdle), which are also presented in Table 2. We will do so by again considering different types of actors.

On the *researcher level*, we argue that (accessible) examples of mixed methods research offer tools and inspiration to researchers to make use of similar designs. This was one of the concrete aims of this Special Issue (Geurts, Davids, and Spierings 2025), and our inventory in Table 1 illustrates that examples of various mixed methods endeavours are already out there. In addition, several articles in this issue offer additional tools and reflections of doing mixed methods research (e.g. Azabar and Thijssen 2025; Mazzucato 2025). Thinking out loud, we can imagine that having a database with examples, thematically clustered or clustered by design, may further spark designing MMR. Similarly, a collaboration database may be fruitful to easily get in touch with colleagues who are interested in setting up a mixed methods research in EMS (e.g. the IMISCOE network may be a nice place to facilitate such collaboration spaces), with scholars realising that MMR is not something that has to be executed alone.

Specifically among PhD scholars, we recognise and conclude that additional support is necessary. The need for proper training – either to learn about mixed methods research, how to collaborate in a mixed methods research or the practicalities of doing it – is high and should be facilitated. For instance, PhD scholars should be able to join workshops and summer schools that are already organised on this topic.^14^ More generally, PhDs benefit from conducting research in an open atmosphere that applauds collaboration and in which there is space to learn new methodological approaches and network with researchers using different methods. Supervisors play a key role here.

On the *journal level*, we would recommend making explicit to what extent journals are open to (and have editors with expertise in) mixed methods research. Similarly, it is helpful if journals indicate to what extent using more words is allowed, and whether using a MMR design is seen as a valid reason to increase the word count or make more extensive use of online appendices. We, moreover, encourage editors to take their responsibility in this process, for example by specifically inviting reviewers who have mixed methods experience or by instructing reviewers who have stated to have expertise in one method to not evaluate the (methodological) part of the manuscript that they do not have expertise in. This would require active involvement of the editors, also in case of unfair comments on the mixed methods design by reviewers, and possibly by making separate guidelines for reviewers on how to consider trade-offs made in using extra words to describe the mixed methods design. Finally, we would encourage journals to recommend and support project-based research notes where prior mono-method articles are embedded in. Doing so makes MMR projects more visible and stimulates proper integration of methods to answer an overarching research question.

With respect to the structural (and more epistemological) context, vaulting poles may need to be a bit longer and this requires efforts from all of us in the academic community. Sharing (both qualitative and quantitative) data that have been already collected openly within the academic community could be one step forward in facilitating mixed methods research. Although qualitative data specifically is seen as more difficult to reuse than quantitative data, we believe sharing these – including reflections of the fieldwork and the authors, situating the data collection – can offer a range of opportunities with respect to mixed methods designs. And, in particular, quantitative data sources could include information on the extent to which subsequent data collection within or outside of the associated research team is possible to enable nested mixed methods designs (e.g. Geurts 2022). Sharing data more would, furthermore, underscore that MMR does not necessarily require one researcher to do it all.

In addition, for mixed methods research to thrive, we need respect from various methodological corners and knowledge that specific research questions benefit from different methods used. We believe that concrete examples illustrating the benefits of mixed methods research is one way forward, as well as facilitating conversations between researchers who use different methodologies on similar themes, with a core goal (or assignment, if needed) to learn from each other.

## 6. Closure

In this manuscript, we pursued three aims using various data sources and analytical approaches. Let us get back to them and share the respective take away messages, if only for closure.

First, we obtained insights into how mixed methods research is generally defined, at least within ethnic and migration studies. The predominant notion is that mixed methods research requires mixing qualitative and quantitative studies, but some argue that also a mix of different quantitative or different qualitative methods can be defined as such. Bringing together the results of different methods is generally considered crucial to call something mixed methods (cf. multimethod). Both the diversity and broadness of the definitions illustrate that mixed methods research can take various shapes, which is the first message we take away from this study. This is particularly important in light of the hurdles, as trying mixed methods research, therefore, does not have to be out of the left field; it can start small by combining methods within one’s (quantitative or qualitative) training or brainstorming with someone who has interest in joining forces based on each respective methodological expertise.

Second, we explored to what extent mixed methods research finds its way into journals. While we argued that ethnic and migration studies are, likely, relatively welcoming to mixed methods research, the share of articles that uses MMR (2020–2022) is on average 3.5 percent, while it seems to be on the rise. At the same time, journals seem to have lowered their word count over time, which is counterproductive; practical issues like this do present hurdles to MMR. We should also stress that, especially, early career scholars expressed interest in using mixed methods designs. However, (perceptions of) the existing hierarchy within departments and what kind of knowledge is valued seem to prevent (junior) scholars from developing such interests and skills. Overcoming all these hurdles requires various actors and norms within the academic community to change, but they do not need to change all at once. In conclusion, we take away that despite the numerous and sturdy hurdles present, there are many different smaller and larger vaulting poles we can take up to slowly but surely face these hurdles.

We already referred to our final aim a lot: inventorying the barriers to conducting mixed methods research, in order to find ways to overcome these. We conclude that conducting mixed methods research is a proper hurdling race with barriers present at many different stages and levels of the race – from formulating an idea via conducting the research to publishing it – whereby the individual is embedded in a network and structure, and this all interlinks. Reasons for not doing mixed methods are practical and tactical, but in many of these motives, assumptions about ‘the other side’, reflecting the definition that mixed methods research involves a quantitative and qualitative pole, play a big part. Often, it is assumed rather than experienced that publishing mixed methods research takes more resources (i.e. time, energy, skills and money). Within the current academic world, this can be understood; however, from this research, we also conclude that numerous researchers see great merit in mixed methods research, and also, these researchers might be your future employers. A 400 metre sprint is finished quicker than a 400 metre hurdle race, but when you like hurdling, rest assures that there is demand for people who can do that too: those academics who want to further our understanding of various messy problems and boost our curiosity in tackling tricky research questions.

At the same time, we collected and presented many options to make jumping the hurdles a bit easier. We sincerely hope that these suggestions are seriously considered by all actors involved, particularly gatekeepers like journal editors, reviewers and supervisors, and that this leads to systemic change in the end. A final take-away message is that you may occupy various roles in which your view and actions on mixed methods research carry weight, whether this is having an open mind to your PhD’s research idea or enabling a higher word count for mixed methods papers as an editor. By sharing our experiences as well as resources (including places to find inspiration), we also aim to support and further the trend bottom up. We hope to facilitate scholars who have questions that beg mixed methods research.

In all of the above, there are few simple truths and lots of inferences. As said, this contribution is a part of the discussion. We would already be rather happy – not satisfied – if each of us (yes, you as a reader too) reflects on our own views and practices, and how these translate to future researchers in ethnic and migration studies (and beyond). Particularly, we invite journals and supervisors to critically reflect on how their norms, rules and preferences might discourage MMR while they do not intend to do so and whether their risk assessment can be validly changed building in valves and safety nets.

## Notes

1 A keen reader might notice that the hope we formulated here does assume a notion of what *we* consider mixed methods research to be. We have ideas on this for sure, but they are in constant flux and we probably do not agree a full 100% with each other. However, at this point, we agree on it implying that (a) the methods or outcomes of applying different methods are really combined when drawing conclusions, (b) the elements being mixed are based on two different data sources, and (c) the methods applied are distinctly different methodologically, while/thus we do not exclude work that mixes two different types of quantitative or qualitative analyses. Clearly, our common ground includes a lot of grey, but the issues discussed in this contribution seem rather independent of these very specific points we could quarrel on. Still, we welcome a lively debate on them, particularly if concrete and tangible consequences on the hurdles *and* vaulting poles are part of that debate and not just abstract generalisms.

2 More detailed and methodological information is given in the text (or footnotes) where relevant.

3 Extensive notes were taken through every keynote and workshop by David de Kort and Matthijs Gillissen, for which we thank them. A special thanks to David, Linda Sloane (RUNOMI) and Laura Cleton (DAMR) for their practical support and input on the organization of the conference.

4 The RU Ethical Commission Social Sciences concluded the survey fulfilled with the criterion for this kind of research (ECSW-LT-2022-9-27-45054).

5 Actually, there is a 0.2 percentage point difference according to the Federal Statistical Office in Germany: 3.7% of the German population has an immigration background with Turkish citizenship (i.e. the so-called ‘first-generation migrants’).

6 And ‘qualitative and quantitative’; both can come first depending on the design. This holds throughout, but this is the last time we add this. You get the point.

7 We also compared the year of obtaining one’s PhD and whether academics conduct MMR based on our survey data. This showed that those who obtained their PhD longer ago were more likely to conduct MMR. This went completely against what we derived from the focus group and conferences we organized, but on second thought does make sense. Our survey asks whether one has *ever* conducted MRR. Those with a longer career are also more likely to say ‘yes’, and this might even be based on a MMR PhD thesis they supervised. Moreover, thinking about it one could even argue that asking the question for ‘the last three years’ would still lead to an inverse relation, if doing MMR is considered more high risk, which links to the type of position one holds. But now, we are getting ahead of ourselves; more on this in Sections 4 and 5.

8 14 QQ; 3 (also) qualitative mixed

9 4 report to have published the research in monographs/edited volumes and 7 in journals; 3 thus published in both. 1 specifically refers to government reports; which is not included in this count. 7 of the respondents who reported to have conducted MMR did not report having published MM work.

10 See: https://help.altmetric.com/support/solutions/articles/6000233311-how-is-the-altmetric-attention-score-calculated-

11 In the survey, we ask for reasons why respondents themselves do not conduct MMR and, more generally, what barriers they think exist.

12 We particularly liked the idea of breaking through or demolishing walls as an analogy. Overcoming hurdles, on the other hand, is managing to get over them, which leaves the hurdle intact. This seems more like reality, but by no means does our choice for hurdles implies we argue against smashing some of these hurdles to pieces.

13 For very narrow discipline-based calls, the risk might be more prominent.

14 E.g. the summer course in Lugano, Switzerland taught by Max Bergman, the GESIS summer course taught by Ingo Rohlfing, the Essex summer course taught by Daphne Watkins, or the Radboud MethodsNet summer course taught by Erin Jenne.

## Appendices

### Appendix A: survey questionnaire

Questionnaire for mixed methods research in migration studies.

Mixed methods are regularly being debated across disciplines and coffee corners. Our experience is that many interesting arguments and views come up in those debates. But not every one speaks up all the time. Via this brief survey (∼5 min), we hope to get a grasp of the scope of views and arguments. The questionnaire hopefully contributes to a publication on the issue, which we can distribute to those who are interested. The survey itself is anonymous and e-mail addresses will be saved separately from your answers, all on secured servers. Thank you for your consideration to participate.

1. How would you define mixed methods research (the first that comes to mind, works for us)?

**Table:**

2a. Have your ever conducted mixed methods research?

**Table:**

No
Yes,

2b. If yes, what methods did you mix?

**Table:**

3. Can you provide your consideration for doing mixed methods research or not doing it? Consideration that hold up for you that is.

**Table:**

Doing
Not doing

4a. Did you publish mixed methods research (i.e. does at least one of your publication include a mixed methods setup)?

**Table:**

No
Yes

4b. If yes, in what journal(s) or with what publisher(s) in the case of books?

**Table:**

5a. What are, according to you, the most important barriers to do and publish mixed methods research? (please explain briefly)

**Table:**

1.
2.
3.
4.
5.
6.

5b. Could you please indicate which one withheld you from doing or publishing mixed methods research?

**Table:**

Withheld me from	DOING	PUBLISHING
1.		
2.		
3.		
4.		
5.		
6.		

6. What else regarding doing mixed methods research would you wanna share with us?

**Table:**

7a. What discipline(s) do you work in?

**Table:**

7b. What is the discipline you formally work in (i.e. your department)?

**Table:**

7c. To what extent would you consider yourself to be an ethnic and migration studies scholar?

**Table:**

0	1	2	3	4	5	6	7	8	9	10
Not at all										very much so

8a. What is your function

**Table:**

PhD student
Postdoctoral researcher
Assistant professor
Associate professor
Full professors
Other: … … … ..

8b. In what year did you defend your PhD thesis?

**Table:**

_ _ _ _
Not yet.
